# Examining the Reaction Times of International Level Badminton Players Under 15

**DOI:** 10.3390/sports6010020

**Published:** 2018-03-05

**Authors:** Mehmet Fatih Yüksel, Gülsen Tosun Tunç

**Affiliations:** 1 Faculty of Education, Necmettin Erbakan University, Konya 42090, Turkey; 2 Faculty of Sports Science, Aksaray University, Aksaray 68100, Turkey; gulsentit@hotmail.com

**Keywords:** badminton, under 15, reaction time

## Abstract

This research was conducted to examine the simple visual and auditory reaction times of badminton players of the national teams and to examine the possible effects of reaction-time average values of badminton players under the age of 15 who participated in the fifth International Rumi Child Sport Games. In total, 48 players (male = 24; female = 24) from six countries (Turkey, Azerbaijan, Bulgaria, Macedonia, Serbia, Georgia) participated in the study. Stature, bodyweight, BMI, dominant and non-dominant hand visual and auditory reaction time values of the participants were detected. At the end of the study, it was determined that there were statistically significant differences between the countries in terms of male dominant and non-dominant hand visual reaction values, and male dominant hand auditory reaction values. It was also determined that there were statistically significant differences between the countries in terms of female bodyweight, BMI, dominant and non-dominant hand visual reaction values, and female non-dominant hand auditory reaction values. There was statistically significant difference between female and male players with regards to dominant and non-dominant hand visual, and non-dominant hand auditory reaction values. In conclusion, it was determined that the reaction times of the top ranking countries in the fifth International Rumi Child Sport Games under-15 were at a better level, and it can be concluded that this factor played an important role for success alongside with technique and tactic features.

## 1. Introduction

Badminton is a racquet sport in which leaps, veers, and quick arm movements are needed [1]. The sport is played by two or four people without physical contact on a rectangle court divided into two equal areas by a net [2]. Badminton is a rapidly developing sport worldwide. There are 188 member countries in the International Badminton Federation, and there are 111 million licensed players around the world [3].

Literature review research indicates that the shuttlecock bounces back 0.93 s after a shot in a rally [4], and that the average time of a rally is in between 6–11.5 s [5,6]. Another study reported that the longest rally times in females and males consecutively were 34.6 and 38.4 s [7]. A different study emphasized that the time of intervals between the points were 27–30 s [8]. Additionally, another study stated that the maximal speed of a shuttlecock was measured as 421 km/h and that it was the fastest ball in the world [9,10]. In another research study, Seth [11] indicated that badminton play was characterized as short period, high intensity, and short intervals. Moreover, coordinative talents are vital in badminton sport. One of these coordinative features is reaction, which is important for responding fast. Particularly the high speed of the shuttlecock leaves too little a time to react, thus, badminton player should quickly and accurately decide during the game. In brief, the fast return of the shuttlecock in less than one second necessitates quick thinking and reacting to the stimulus during the game. 

When the literature was examined, although there were many research studies, which examined the reaction times of the badminton players and stated that they had better reaction times compared to control groups [9,12,13,14,15,16,17], the reaction times of under-15 international level players were not observed. That is why it is evaluated vital to examine the reaction times of the under-15 international badminton players for a contribution to the field.

The purpose of this study was to examine simple visual and auditory reaction times of under-15 badminton players from different countries who participated in the fifth International Rumi Child Sport Games. A secondary purpose was to examine the possible effects of reaction times on tournament results.

## 2. Materials and Methods

### 2.1. Participants

The study was conducted on players of under-15 national teams from 6 countries (Turkey, Azerbaijan, Bulgaria, Macedonia, Serbia, Georgia) who participated in the 5th International Rumi Child Sport Games. Since the six national teams who participated in the tournament were composed of 4 female and 4 male players, 48 players in total participated in the study. This study was conducted according to the Helsinki Declaration.

### 2.2. Place and Time of Measurement

The measurements were taken a day before the game between 10:00 and 12:00 a.m. The measurements were conducted in a room in the sport hall, which was insulated against sound and noise, with moderate light intensity, and cleared of the materials that could disturb the concentration of the players.

### 2.3. Procedures

All participants signed an informed consent form. Permission was gained from the tournament organization committee and the team managers.

In defining the ages of the players, identification and passport information was based on. The stature, body weight, and simple visual-auditory values of the players were recorded after tests and measurements, body mass index (BMI) was calculated by means of the formula.

After the stature and body weight measurements, 15 min of warm-up period was given to the players before the reaction time test. Necessary information about the tests was explained to the players before the applications. They were shown the applications of the tests. They were repeatedly informed about the objective and importance of the research study, thus their motivation and ambition was to be increased. The players participated in the measurements and tests wearing sportswear.

The dominant hands of the players were determined based on the question “With which hand do you hold the racket?”

Height and Body weight: In the linear measurements a tapeline with 0.01 m sensitivity score was used. Weight measurements were made with a digital weighing scale with a sensitivity level of 0.01 kg. The measurements were made twice and the average was taken [18].

Body mass index; using body weights and lengths, BMI was determined using the BMI = Body weight/(Stature in meters)^2^ formula.

Visual–auditory reaction times measurement: visual and auditory reaction times were located via New-Test 2000 measurement device. The device is composed of two parts. The first part is the button that the subject presses with the finger against audible (voice) or visual (light) stimulus. The second part is used by the tester. It manages the type (visual or auditory) and number of the stimuli that are sent to the subject. The subject and the tester sit face to face on a table, and the tester conducts the test. 

The subject is in a sitting position, one hand is on the table in front of the device that emits audible or light stimulus, while the other hand is let free without taking support from anything. In auditory reaction test, the tester gives the test start warning when the subject is ready. From that moment on, an auditory stimulus (“beep” sound) is heard within randomized periods. Hearing the stimulus, the subject presses the button on the device as fast as possible and stops the sound stimulus. In the visual reaction test, the button on the device flashes a light, and the subject presses the button as fast as possible to turn off the light stimulus. The time span between the stimulus and the reaction is called as the reaction time. The subject repeated the measurements for 10 times. The first 5 of them were accepted as exercise, and the average of the last 5 repeats was accepted as the reaction time, and recorded in ms [18].

### 2.4. Statistical Analysis

SPSS 24.0 (IBM Corp., Armonk, NY, USA) program was used in the analysis of the data obtained through the research. Arithmetic averages and standard deviations were given by descriptive statistics. Considering the number of the participant players, Kruskal–Wallis analysis (one of non-parametric tests) was used in order to define whether there was a significant difference between countries regarding parameters. If there was a significant difference, Kruskal–Wallis analysis was also used to locate the countries with significant difference. Mann–Whitney U analysis was used in order to examine whether there was a statistically significant difference in the reaction-time parameter with regards to gender. Significance level was admitted as *p* < 0.05.

## 3. Results

Table 1 shows the age, height, body weight, BMI and simple visual and auditory reaction time average values of country athletes participating in the study.

When Table 2 is examined, according to the Kruskal–Wallis analysis results, it was defined that there was statistically significant differences (*p* < 0.05) between;

- Bulgarian players and Macedonian players for bodyweight,
- Bulgarian players and Turkish/Serbian players for BMI,
- Turkish and Bulgarian players for dominant hand visual reactions,
- Turkish players and Bulgarian players for non-dominant hand visual reactions,
- Serbian players and Bulgarian/Azeri players for non-dominant hand visual reactions,
- Turkish/Serbian/Macedonian players and Bulgarian players for non-dominant hand auditory reactions,
- Serbian players and Azeri players for non-dominant hand auditory reactions.

There was no statistically significant difference between the countries in terms of age, stature, and dominant hand auditory reaction parameters according to the Kruskal–Wallis Analysis results (*p* > 0.05). 

When Table 3 is examined, according to the Kruskal–Wallis Analysis results, it was defined that there was statistically significant differences (*p* < 0.05) between;

- Serbian players and Bulgarian players for dominant hand visual reactions,
- Turkish/Serbian players and Bulgarian players for non-dominant hand visual reactions,
- Serbian players and Bulgarian players for dominant hand auditory reactions.

There was no statistically significant difference between the countries in terms of age, stature, bodyweight, BMI, and non-dominant hand auditory reaction parameters according to the Kruskal–Wallis analysis results (*p* > 0.05). 

When Table 4 is examined, it was defined that there was a statistically significant difference (*p* < 0.05) between female and male groups with regards to three parameters. Firstly, dominant hand visual reactions (U = 164.000, *p* < 0.05), secondly, non-dominant hand visual reactions (U = 137.000, *p* < 0.05), and thirdly, non-dominant hand auditory reactions (U = 107.500, *p* < 0.05) parameters.

## 4. Discussion and Conclusions

In our research, we aimed to examine the simple visual and auditory reaction times of under-15 badminton players of Turkey, Azerbaijan, Bulgaria, Macedonia, Serbia, and Georgia national teams which participated in the fifth International Rumi Child Sport Games. 

There was no statistically significant difference between the countries in terms of average age, and stature parameters of the female and male groups. Considering the height of the badminton net from the ground (152–155 cm), it can be stated that all of the players participating in the research were far taller than the height of the net, so net height did not affect success. The fact that the height of the subject group in the research was higher than the net was supported by numerous similar studies regarding the height of the badminton players and net height [1,9,15,19,20,21,22,23,24].

There was no statistically significant difference between the countries in terms of body weight, and BMI parameters of the male groups (*p* > 0.05). In the country comparison of female badminton players, there were statistically significant differences (*p* < 0.05) between Serbia and Bulgaria for bodyweight. There was also statistically significant difference among Turkish/Serbian and Bulgarian players’ BMI. Body weight has a vital role in badminton, since it requires the body to leap up numerous times from the ground despite gravity. Accordingly, it can be stated that Bulgarian female players’ BMI values were higher than the average, which created a disadvantage. Also, the BMI findings of all the groups were within the normal limits stated by the World Health Organization [25].

When the visual and auditory reaction time results of the female and male players participating in the research were examined, Turkey and Serbia had the lowest (best) values for both dominant and non-dominant hand. Moreover, it was observed that male players compared to females, and dominant hand compared to non-dominant hand had lower (better) values. According to research conducted in India, the visual times of the control group, which was formed by male badminton players and healthy individuals, were respectively 283.46 ms and 347.76 ms. There was statistically significant difference in favor of the badminton players [13]. Remarkable improvement was observed in the visual time values of the badminton players who underwent six-week exercises [14]. According to another research conducted in Iran, the visual reaction values of the male badminton players in dominant and non-dominant hands respectively were determined as 130.46 ms and 131.27 ms [16]. Similarly, in another research study on under-15 male badminton players, it was reported that the visual reaction time was 0.27 s [26]. In a study in Poland on the national badminton players who were visually and aurally stimulated, dominant and non-dominant hand reaction time values were respectively determined as 0.26 s and 0.27 s [9]. Existing literature findings, except the Dube et al. [16] study, are generally supporting our research. 

In research studies on different sport branches (Football, Handball, Volleyball, Wrestling, Ice-skating), simple visual and auditory reaction time average values were reported to be in between 355–405 ms [27,28,29]. However, it was observed that the reaction time average values of this study were better compared to the abovementioned other sport branches. This might be because the reaction time improving exercises were conducted more in badminton sports branch. Additionally, that the badminton players have better reaction times might be explained with the result of long years’ training with racquet. In another study on national badminton players under-17, the results of visual and auditory reaction time values were similar to our research while auditory reaction time values were (better) lower [15]. Similarly, in a research on international players by Revan et al. [12], the auditory reaction values reported were lower compared to visual reaction values. On the other hand, different from above mentioned studies, in our study, it was observed that the visual reaction values of both female and male players in both hands were lower (better) compared to auditory values. This may be because of the variables at the measuring moment. Moreover, that the visual reaction times are more improved compared to auditory reactions is an expected situation for the players who exercised for years reacting to visual stimulus, which is the main characteristic of the badminton game, and this situation is supported with the data obtained.

A player may need to practice for hours, days, or even months to improve oneself. As a result of the developments in training science, it is known that the quality of different exercise methods have been increased and therefore it is reflected on physical performance. It can be accepted as the strength of the study that all of the data obtained from the research was from the international level badminton players with a full participation. Yet, it is considered that further research studies, similar to this narrow-scoped study, should be conducted on different age categories and with broader participation. Thus, the findings of this research will be ascertained whether they are replicable in other samples or cohorts of badminton players. 

In this research, visual and auditory reaction times of badminton players of the national teams under 15, who participated in the fifth International Rumi Child Sport Games from different countries, were examined. Having the lowest (best) reaction time values in both males and females, Turkish and Serbian national teams ranked in the first three in the tournament. During the game it is vital for the players to have a good level of reaction time against heavy hits like smash, drive, and net-kill. We consider that reaction time is an important feature particularly for the badminton players as it is important for many other sports branch. The players of the ranking first three countries having better reaction times supported this determination. Therefore, it can be clearly concluded that the reaction time plays an important role for success.

We evaluate that it is a necessity for the training programs to include reaction time improving exercises considering the characteristic features of badminton such as fast speed of shuttlecock and the stimulus being continuously visual. Thus, it can be stated that the reaction time of the players will improve and it will contribute to the game performance. It is suggested for the trainers/sports scientists to include exercises such as Standing Ball Drop, Ball Drop and Sprint, Kneeling Ball Catch which involve particularly visual stimuli.

## Figures and Tables

**Table 1:** The average values of age, height, weight, BMI and basic reaction times of the athletes.

Parameters	Gender	*n*	TURKEY	AZERBAIJAN	BULGARIA	MACEDONIA	SERBIA	GEORGIA
Age	F	4	14.40 ± 0.29	14.68 ± 0.22	14.58 ± 0.26	14.2 ± 0.43	14.43 ± 0.28	14.43 ± 0.38
	M	4	14.50 ± 0.22	14.33 ± 0.30	14.48 ± 0.40	14.28 ± 0.33	14.43 ± 0.30	14.30 ± 0.22
Height	F	4	171.0 ± 5.35	165.75 ± 3.50	165.75 ± 5.97	160.0 ± 6.38	167.25 ± 7.63	167.75 ± 4.03
	M	4	175.25 ± 2.50	167.25 ± 5.62	167.25 ± 8.85	169.0 ± 5.48	170.0 ± 3.56	167.50 ± 2.50
Weight	F	4	52.25 ± 7.59	55.90 ± 4.44	61.75 ± 4.65	49.63 ± 2.06	51.25 ± 4.65	54.50 ± 3.98
	M	4	61.25 ± 2.75	58.38 ± 4.04	56.75 ± 7.18	63.50 ± 5.0	55.25 ± 2.50	61.33 ± 3.62
BMI	F	4	17.81 ± 1.93	20.33 ± 1.07	22.47 ± 1.12	19.41 ± 0.86	18.35 ± 1.72	19.87 ± 1.78
	M	4	19.96 ± 1.21	20.87 ± 1.16	20.23 ± 1.11	22.26 ± 1.85	19.16 ± 1.54	21.85 ± 0.88
Visual reaction dominant hand	F	4	230.25 ± 8.38	309.75 ± 25.89	326.75 ± 20.11	294.75 ± 20.32	261.75 ± 29.02	303.75 ± 20.89
	M	4	228.25 ± 13.87	284.50 ± 33.93	300.50 ± 35.52	268.0 ± 25.81	224.0 ± 23.93	276.75 ± 25.83
Visual reaction non-dominant hand	F	4	320.25 ± 14.57	354.75 ± 9.74	356.0 ± 6.16	340.0 ± 9.42	306.0 ± 27.26	342.50 ± 9.26
	M	4	293.50 ± 11.09	323.0 ± 12.25	352.0 ± 10.86	312.0 ± 11.86	271.25 ± 21.38	320.0 ± 7.48
Auditory reaction dominant hand	F	4	296.75 ± 20.11	317.25 ± 23.54	319.75 ± 18.63	307.50 ± 20.24	287.0 ± 23.74	319.50 ± 18.81
	M	4	271.0 ± 28.72	314.25 ± 24.87	329.25 ± 16.60	299.0 ± 20.94	260.50 ± 33.32	312.50 ± 17.71
Auditory reaction non-dominant hand	F	4	312.25 ± 20.32	338.0 ± 17.57	347.25 ± 18.61	321.0 ± 11.63	305.50 ± 23.02	330.25 ± 9.67
	M	4	286.50 ± 23.45	309.75 ± 21.20	316.50 ± 21.46	300.25 ± 13.30	282.50 ± 26.94	307.50 ± 11.68

**Table 2:** The Kruskal–Wallis analysis results of the female badminton players based on countries.

Parameters	Country	*n*	Mean Rank	df	*p*	Difference
Age	TURKEY (T)	4	11.00			
	AZERBAIJAN (A)	4	17.50			
	BULGARIA (B)	4	15.13			
	MACEDONIA (M)	4	7.88	5	0.468	
	SERBIA (S)	4	11.63			
	GEORGIA (G)	4	11.88			
	Total	24				
Height	TURKEY (T)	4	18.63			
	AZERBAIJAN (A)	4	11.63			
	BULGARIA (B)	4	12.75			
	MACEDONIA (M)	4	6.00	5	0.245	-
	SERBIA (S)	4	14.00			
	GEORGIA (G)	4	12.00			
	Total	24				
Weight	TURKEY (T)	4	11.38			
	AZERBAIJAN (A)	4	15.38			
	BULGARIA (B)	4	20.88			
	MACEDONIA (M)	4	5.25	5	0.041 *	M < B
	SERBIA (S)	4	9.00			
	GEORGIA (G)	4	13.13			
	Total	24				
BMI	TURKEY (T)	4	6.00			
	AZERBAIJAN (A)	4	14.75			
	BULGARIA (B)	4	22.00			
	MACEDONIA (M)	4	11.75	5	0.021 *	T < B
	SERBIA (S)	4	7.25			S < B
	GEORGIA (G)	4	13.25			
	Total	24				
Visual reaction dominant hand	TURKEY (T)	4	3.25			
	AZERBAIJAN (A)	4	16.63			
	BULGARIA (B)	4	19.75			
	MACEDONIA (M)	4	13.00	5	0.010 *	T < B
	SERBIA (S)	4	7.25			
	GEORGIA (G)	4	15.13			
	Total	24				
Visual reaction non-dominant hand	TURKEY (T)	4	5.75			
	AZERBAIJAN (A)	4	19.25			
	BULGARIA (B)	4	20.50			T < B
	MACEDONIA (M)	4	11.63	5	0.003 *	S < B
	SERBIA (S)	4	4.25			S < A
	GEORGIA (G)	4	13.63			
	Total	24				
Auditory reaction dominant hand	TURKEY (T)	4	9.38			
	AZERBAIJAN (A)	4	14.75			
	BULGARIA (B)	4	16.13			
	MACEDONIA (M)	4	11.00	5	0.297	-
	SERBIA (S)	4	7.13			
	GEORGIA (G)	4	16.63			
	Total	24				
Auditory reaction non-dominant hand	TURKEY (T)	4	8.25			
	AZERBAIJAN (A)	4	16.50			T < B
	BULGARIA (B)	4	19.75			S < B
	MACEDONIA (M)	4	9.88	5	0.044 *	M < B
	SERBIA (S)	4	5.88			S < A
	GEORGIA (G)	4	14.75			
	Total	24				

* *p* < 0.05.

**Table 3:** The Kruskal–Wallis analysis results of the male badminton players based on countries.

Parameters	Country	*n*	Mean Rank	df	*p*	Difference
Age	TURKEY (T)	4	15.50			
	AZERBAIJAN (A)	4	11.13			
	BULGARIA (B)	4	14.63			
	MACEDONIA (M)	4	9.75	5	0.812	-
	SERBIA (S)	4	13.50			
	GEORGIA (G)	4	10.50			
	Total	24				
Height	TURKEY (T)	4	20.75			
	AZERBAIJAN (A)	4	9.75			
	BULGARIA (B)	4	11.13			
	MACEDONIA (M)	4	12.38	5	0.165	-
	SERBIA (S)	4	12.88			
	GEORGIA (G)	4	8.13			
	Total	24				
Weight	TURKEY (T)	4	15.88			
	AZERBAIJAN (A)	4	11.63			
	BULGARIA (B)	4	9.13			
	MACEDONIA (M)	4	17.50	5	0.128	-
	SERBIA (S)	4	5.50			
	GEORGIA (G)	4	15.38			
	Total	24				
BMI	TURKEY (T)	4	9.50			
	AZERBAIJAN (A)	4	12.75			
	BULGARIA (B)	4	10.13			
	MACEDONIA (M)	4	18.00	5	0.056	-
	SERBIA (S)	4	5.63			
	GEORGIA (G)	4	19.00			
	Total	24				
Visual reaction dominant hand	TURKEY (T)	4	6.13			
	AZERBAIJAN (A)	4	16.50			
	BULGARIA (B)	4	19.75			
	MACEDONIA (M)	4	12.50	5	0.017 *	S < B
	SERBIA (S)	4	5.00			T < B
	GEORGIA (G)	4	15.13			
	Total	24				
Visual reaction non-dominant hand	TURKEY (T)	4	6.25			
	AZERBAIJAN (A)	4	15.88			
	BULGARIA (B)	4	22.38			
	MACEDONIA (M)	4	12.00	5	0.002 *	T < B
	SERBIA (S)	4	3.25			S < B
	GEORGIA (G)	4	15.25			
	Total	24				
Auditory reaction dominant hand	TURKEY (T)	4	6.50			
	AZERBAIJAN (A)	4	16.13			
	BULGARIA (B)	4	19.75			
	MACEDONIA (M)	4	12.13	5	0.017 *	S < B
	SERBIA (S)	4	4.75			
	GEORGIA (G)	4	15.75			
	Total	24				
Auditory reaction non-dominant hand	TURKEY (T)	4	9.63			
	AZERBAIJAN (A)	4	14.63			
	BULGARIA (B)	4	16.88			
	MACEDONIA (M)	4	11.50	5	0.439	-
	SERBIA (S)	4	7.75			
	GEORGIA (G)	4	14.63			
	Total	24				

* *p* < 0.05.

**Table 4:** The averages of reaction times and Mann–Whitney U analysis results of the female and male badminton players.

Parameters	Gender	*n*	Mean	Std. Deviation	Mean Rank	U	*p*
Visual reaction dominant hand	F	24	287.83	38.27	19.33	164.000	0.011 *
	M	24	263.67	37.74	29.67		
Visual reaction non-dominant hand	F	24	336.58	22.47	18.21	137.000	0.002 *
	M	24	311.96	28.20	30.79		
Auditory reaction dominant hand	F	24	307.96	22.45	22.33	236.000	0.283
	M	24	297.75	33.03	26.67		
Auditory reaction non-dominant hand	F	24	325.71	21.34	16.98	107.500	0.000 *
	M	24	300.50	22.01	32.02		

* *p* < 0.05.
